# Molecular mechanisms detected in yak lung tissue via transcriptome-wide analysis provide insights into adaptation to high altitudes

**DOI:** 10.1038/s41598-021-87420-7

**Published:** 2021-04-08

**Authors:** Qianyun Ge, Yongbo Guo, Wangshan Zheng, Shengguo Zhao, Yuan Cai, Xuebin Qi

**Affiliations:** 1 College of Animal Science and Technology, Gansu Agricultural University, Lanzhou, 730070 China; 2 State Key Laboratory of Genetic Resources and Evolution, Kunming Institute of Zoology, Chinese Academy of Sciences, Kunming, 650223 China

**Keywords:** Gene expression, Gene regulation

## Abstract

Due to their long-term colonization of and widespread distribution in plateau environments, yaks can serve as an ideal natural animal model for the adaptive evolution of other plateau species, including humans. Some studies reported that the lung and heart are two key organs that show adaptive transcriptional changes in response to high altitudes, and most of the genes that show differential expression in lung tissue across different altitudes display nonlinear regulation. To explore the molecular mechanisms that are activated in yak lung tissue in response to hypoxia, the mRNAs, lncRNAs and miRNAs of lung tissue from 9 yaks living at three different altitudes (3400 m, 4200 m and 5000 m), with three repetitions per altitude, were sequenced. Two Zaosheng cattle from 1500 m were selected as low-altitude control. A total of 21,764 mRNAs, 14,168 lncRNAs and 1209 miRNAs (305 known and 904 novel miRNAs) were identified. In a comparison of yaks and cattle, 4975 mRNAs, 3326 lncRNAs and 75 miRNAs were differentially expressed. A total of 756 mRNAs, 346 lncRNAs and 83 miRNAs were found to be differentially expressed among yaks living at three different altitudes (fold change ≥ 2 and P-value < 0.05). The differentially expressed genes between yaks and cattle were functionally enriched in long-chain fatty acid metabolic process and protein processing, while the differentially expressed genes among yaks living at three different altitudes were enriched in immune response and the cell cycle. Furthermore, competing endogenous RNA (ceRNA) networks were investigated to illustrate the roles of ceRNAs in this process, the result was also support the GO and KEGG analysis. The present research provides important genomic insights for discovering the mechanisms that are activated in response to hypoxia in yak lung tissue.

## Introduction

The organic mechanisms that mediate adaptation to high-altitude environments have attracted widespread attention in recent years. The genomes of some mammals that inhabit highlands, including human highlanders, have been sequenced, and many genes are associated with adaptation to high altitudes^1–9^. Focusing on the mechanisms underlying transcriptomic changes can provide insights into the adaptive evolution of other plateau species, including humans. Due to their long-term colonization of and widespread distribution in the plateau, Tibetans and yaks are two ideal models for studying the ability to adapt to plateau environments^5,10,11^. Compared with those of lowland cattle, yak lungs have developed physiological characteristics that are adapted to high-altitude hypoxia, including a larger pulmonary alveolar area per unit area, a thinner alveolar septum, a thinner blood–air barrier and smooth muscles within the arteriole wall of the microartery with a diameter of < 50 mm, whereas lowland cattle do not have such a structure. These physiological features can promote more efficient blood flow for the transport of oxygen under hypobaric hypoxia. The lung is a central functional organ in the respiratory system, and it plays a substantial role in adaptation to hypoxia in plateau environments. In addition to allowing an animal's body to adapt to external environmental stimuli through a series of physiological changes, gene expression, as an intermediate phenotype linking DNA sequences and physiological traits, plays an important role in revealing molecular pathways/networks associated with genetic adaptation^12,13^. There is growing evidence that changes in gene expression are also essential for adaptation to high altitudes^14–16^.

Some studies investigated the differences in gene expression in the heart between yaks and cattle and reported that some genes associated with the oxygen supply system and the defense systems that respond to hypoxic conditions are differentially expressed^17^. Further study also revealed that the lung and heart are two key organs that show adaptive transcriptional changes in response to high altitudes, and most of the genes that are differentially expressed in the lung tissue across different altitudes display nonlinear regulation^18^. To identify organic mechanisms that mediate adaptation of the lung tissue to hypoxia in yaks, we conducted transcriptome analysis of lung tissue responses to hypoxia in yaks and cattle. To further study the changes in gene regulation that occur in response to hypoxia at increasing altitudes, we collected lung tissue samples from 9 yaks with long-term residence at three different altitudes (3400 m, 4200 m, and 5000 m), three repetitions per altitude. Since yaks are a native plateau species, they are only distributed between 3000 and 5000 m above sea level, so there is no lowland control. In phylogeny, the taurine cattle (*Bos taurus*) is the lowland bovine species that is most closely related to yaks and can be used as a lowland control^19^. We chose two Zaosheng cattle, China's native species, as a lowland control that lives at 1500 m and is geographically adjacent to the Tibetan Plateau. We performed RNA sequencing (RNA-Seq) and mapped the detailed transcriptome of lung tissue. The findings of this study improve our understanding of the molecular mechanisms that are activated in response to hypoxia and are useful for developing strategies to improve the adaptation of other plateau species.

## Results

### Sequencing and mapping of mRNAs and lncRNAs

Transcriptome data sets were generated by RNA-seq to develop a comprehensive catalogue of mRNAs and lncRNAs from the bovine lung. Illumina sequencing of bovine lung tissues yielded a total of 932.336 M raw reads. The Phred Quality Score of the samples was more than 91.7% for Q30. The clean data included 83.501 M reads on average. Subsequently, the clean reads were mapped onto the bovine reference genome using HiSAT2^20^. The average mapping rate of the eleven samples was 86.28%, and the unmapped rates were between 8.51% and 37.01%. The clustering of samples based on the variations in the gene expression was shown in Figure S1. A detailed summary of the sequencing results is shown in Table S1; the results indicated that the transcriptome sequencing data were of high quality and had a suitable mapping rate (Figure S2).

### Identification of miRNAs

Eleven small RNA libraries were constructed and sequenced independently to identify miRNAs in yak and cattle lung tissue. A total of 109.949 M raw reads were generated, and 100.664 M clean reads were obtained, which accounted for more than 92% of the raw data. Among eleven individual libraries, the minimum rates of removed reads were at a suitable level, and the Q30 values of the raw or clean data were high enough to indicate high-quality small RNA sequencing (Table S2). The length of most sequences was distributed in the range of 21.7–22.2 nt, and the highest percentage was observed for a length of 22 nt, which is consistent with the typical size of miRNAs (Figure S2). In total, 305 known and 904 novel miRNAs were identified using miRBase (Release 21) and miRDeep2^21^ (Table S3).

### Identification of differentially expressed mRNAs, lncRNAs and miRNAs

**Figure 1:**
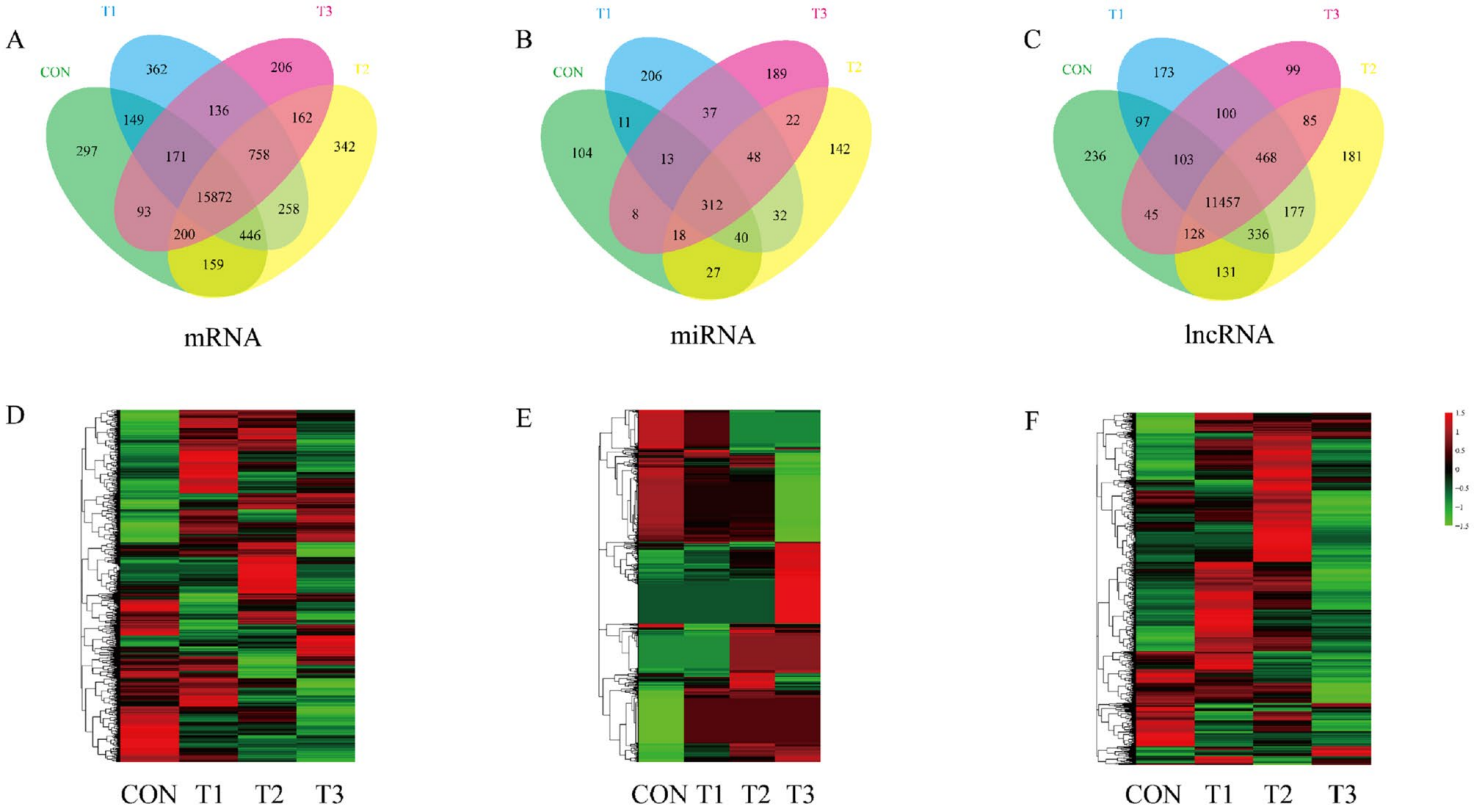
Venn analysis of mRNAs (**A**), miRNAs (**B**) and lncRNAs (**C**) detected among four groups. Cluster analysis of expression of mRNAs (**D**) miRNAs (**E**) and lncRNAs (**F**). The data are depicted as matrices in which each row represents one mRNA, miRNA, or lncRNA and each column represents one of the samples. Relative mRNA, miRNA, or lncRNA expression is depicted according to the color scale shown at the top. Red and green represent high and low relative expression, respectively. The magnitude of deviation from the median is represented by color saturation.

The identification of differentially expressed mRNAs, lncRNAs and miRNAs was performed using edgeR with a threshold of fold change ≥ 2 and P-value < 0.05. A higher number of differentially expressed mRNAs, miRNAs and lncRNAs was obtained from the CON/T1, CON/T2 and CON/T3 pairwise comparisons than from the T1/T2, T1/T3 and T2/T3 pairwise comparisons (Tables 1, 2). A total of 4975 mRNAs, 3326 lncRNAs and 75 miRNAs were found to be differentially expressed between the yak and cattle groups. A total of 756 mRNAs, 346 lncRNAs and 83 miRNAs were found to be differentially expressed among the yaks living at three different altitudes. The details and distribution about differentially expressed mRNAs, miRNAs and lncRNAs in each pairwise comparison were shown in Table 1. Cluster analysis of differentially expressed mRNAs, miRNAs and lncRNAs was conducted with a heatmap (Fig. 1). Then, these differentially expressed noncoding RNAs (ncRNAs) were intersected with predicted target genes. As a result, 13,283 and 3673 genes targeted by miRNAs and lncRNAs, respectively, were screened out (Tables S4 and S5).

**Table 1:** Differentially expressed mRNAs, lncRNAs and miRNAs.

Contrasts	mRNA		miRNA		lncRNA	
	Up	Down	Up	Down	Up	Down
CON VS T1	1011	1019	19	12	740	713
CON VS T2	779	703	5	5	489	454
CON VS T3	691	772	16	18	454	476
T1 VS T2	117	158	11	11	46	84
T1 VS T3	120	160	6	11	48	95
T2 VS T3	119	82	23	21	48	25

ZSlg01 and ZSlg02 indicate libraries derived from the lung tissue of Zaosheng cattle in two biological replicates belonging to the CON group.

MQlg01, MQlg02, and MQlg03 indicate libraries derived from the lung tissue of yaks living at an altitude of 3,400 m in three biological replicates belonging to the T1 group.

LZlg01, BGlg02, and DXlg03 indicate libraries derived from the lung tissue of yaks living at an altitude of 4,200 m in three biological replicates belonging to the T2 group.

ADlg01, ADlg02, and ADlg03 indicate libraries derived from the lung tissue of yaks living at an altitude of 5,000 m in three biological replicates belonging to the T3 group.

**Table 2:** Characteristics of samples.

Sample	Category	Sample size	Sampling site	Altitude (m)	Duplicates
CON	Zaosheng cattle	2	Ningxian, Gansu, China	1500	ZSlg01
					ZSlg02
T1	Yak	3	Maqu, Gannan Tibetan Autonomous Prefecture, Gansu, China	3400	MQlg01
					MQlg02
					MQlg03
T2	Yak	3	Dangxiong, Linzhou and Bange, Tibet Autonomous Region, China	4200	LZlg01,
					BGlg02
					DXlg03
T3	Yak	3	Anduo, Tibetan Autonomous Region, China	5000	ADlg01
					ADlg02
					ADlg03

### Altitude-related transcriptomic profiles

We collected nine lung samples in total with three from each of the three elevations. In total, 275 genes showed significant differential expression between the yak lung samples from the T1 and T2 groups, and 280 and 201 differentially expressed genes (DEGs) were identified from the comparisons of the T1 and T3 groups and the T2 and T3 groups, respectively. We performed a STEM analysis to group the DEGs of yaks at three different altitudes on the basis of expression pattern. With an FC threshold of 2, the number of DEGs was too low to identify the enriched terms, and we thus adjusted the FC threshold to 1.5. Using this criteria, 3060 genes were partitioned into four significantly (*P* < 0.05) enriched altitude profiles (Figure S3). The dominance of the cross-altitude differentially expressed genes in lung display a nonlinear regulation, implying a generally nonlinear correlation between expression level and elevation in lung.

### Functional enrichment analyses of differentially expressed ncRNA target genes and differentially expressed mRNAs

To investigate the functions of differentially expressed lncRNAs and miRNAs, enrichment analyses of their target genes were performed. In a comparison of yaks and cattle, for target genes of the differentially expressed miRNAs (Table S6), metabolic pathways were significantly enriched, including glycerophospholipid metabolism (KO00564), oxidative phosphorylation (KO00190) and glycosaminoglycan biosynthesis-heparan sulfate/heparin (KO00534). For target genes of differentially expressed lncRNAs (Table S7), metabolic pathways were also significantly enriched, including metabolism of xenobiotics by cytochrome P450 (KO00980), drug metabolism-cytochrome P450 (KO00982), drug metabolism-other enzymes (KO00983), retinol metabolism (KO00830), ascorbate and aldarate metabolism (KO00053), pentose and glucuronate interconversion (KO00040) and steroid hormone biosynthesis (KO00140). However, the immune system was significantly enriched for the target genes of the differentially expressed miRNAs among the yaks living at three different altitudes (Table S8), including leukocyte transendothelial migration (KO04670), hematopoietic cell lineage (KO04640) and T cell receptor signaling pathway (KO04660). Environmental information processing, such as the FoxO signaling pathway (KO04068), cell adhesion molecules (CAMs) (KO04514) and the sphingolipid signaling pathway (KO04071), was also highly represented. Cellular processes, such as apoptosis (KO04210) and the p53 signaling pathway (KO04115), were also highly represented. For target genes of differentially expressed lncRNAs (Table S9), organismal systems were significantly enriched, including pancreatic secretion (KO04972), renin secretion (KO04924), Toll-like receptor signaling pathway (KO04620), complement and coagulation cascades (KO04610), circadian rhythm (KO04710) and cardiac muscle contraction (KO04260). Cellular processes were also significantly enriched, including gap junctions (KO04540), adherens junctions (KO04520) and tight junctions (KO04530). These results suggested that abnormal ncRNA expression may affect metabolism, the immune system, cellular processes and environmental information processing in the bovine lung by regulating target genes.

**Figure 2:**
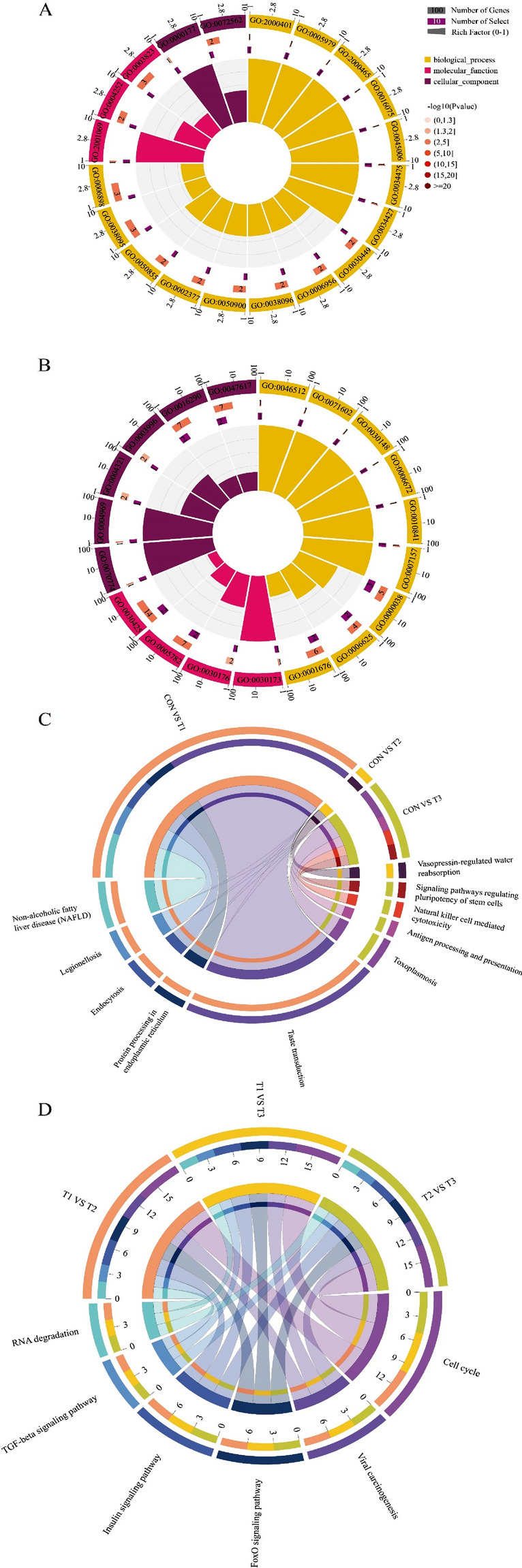
The function of differentially expressed mRNAs was analyzed by KEGG and GO enrichment. GO enrichment of comparison between yak and cattle (**A**) and comparison among yaks living at three different altitudes (**B**). The results from the enrichment analysis are presented using an enrichment cycle diagram. Four circles were included from the outside to the inside. The first circle indicates the enrichment classification. The number of genes (scale) is shown outside of the circle. Different colors represent different categories. The second circle shows the number of background genes in the categories and their Q or *P* values. A longer bar indicates a higher number of genes, and a redder color indicates a smaller value. The third circle shows the total number of differentially expressed genes in the categories. The fourth circle presents the RichFactor value of each category (the number of differentially expressed genes in this category divided by the number of background genes). Each small grid of auxiliary background lines represents 0.1. Analysis of significantly enriched Kyoto Encyclopedia of Genes and Genomes (KEGG) pathways of comparison between yak and cattle (**C**) and comparison among yaks living at three different altitudes (**D**). The significantly enriched KEGG pathways had *P* values < 0.05. Each line represents a gene, and the number of lines indicates the enriched genes. GO and KEGG analysis was visualized by the OmicShare tools, a free online platform for data analysis (http://www.omicshare.com/tools).

As the expression of mRNAs is directly related to biological characteristics, the function of differentially expressed mRNAs was analyzed by KEGG and GO enrichment. When we compared yaks with cattle, differentially expressed mRNAs were classified into three major functional categories (‘biological processes’, ‘molecular function’, and ‘cellular components’) and were enriched in 563 GO categories. The top 10 included seven in “biological process”, two in “molecular function” and one in “cellular components”, of which ‘long-chain fatty acid metabolic process’ and ‘protein targeting to peroxisome’ terms were most significant (Fig. 2, Table S10). Differentially expressed mRNAs were enriched in 62 GO categories among yaks living at three different altitudes. The top 10 included 8 in “biological process”, one in “molecular function” and one in “cellular components”, of which the ‘immune response’ term was most significant (Fig. 2, Table S11). We also performed KEGG pathway enrichment analyses to evaluate the biological significance of the DEGs. A hypergeometric test with a P-adjusted value cutoff of 0.05 was used as the criterion for pathway detection (Fig. 2). To confirm the gene expression patterns, some mRNAs and lncRNAs were randomly selected to be validated by q-PCR. The results were in concordance with the RNA-seq data (Figure S4). RT-qPCR was performed with the internal control β-actin using the 2^−ΔΔCt^ method.

### Differential RNA expression identify core regulatory networks & RNAs

**Figure 3:**
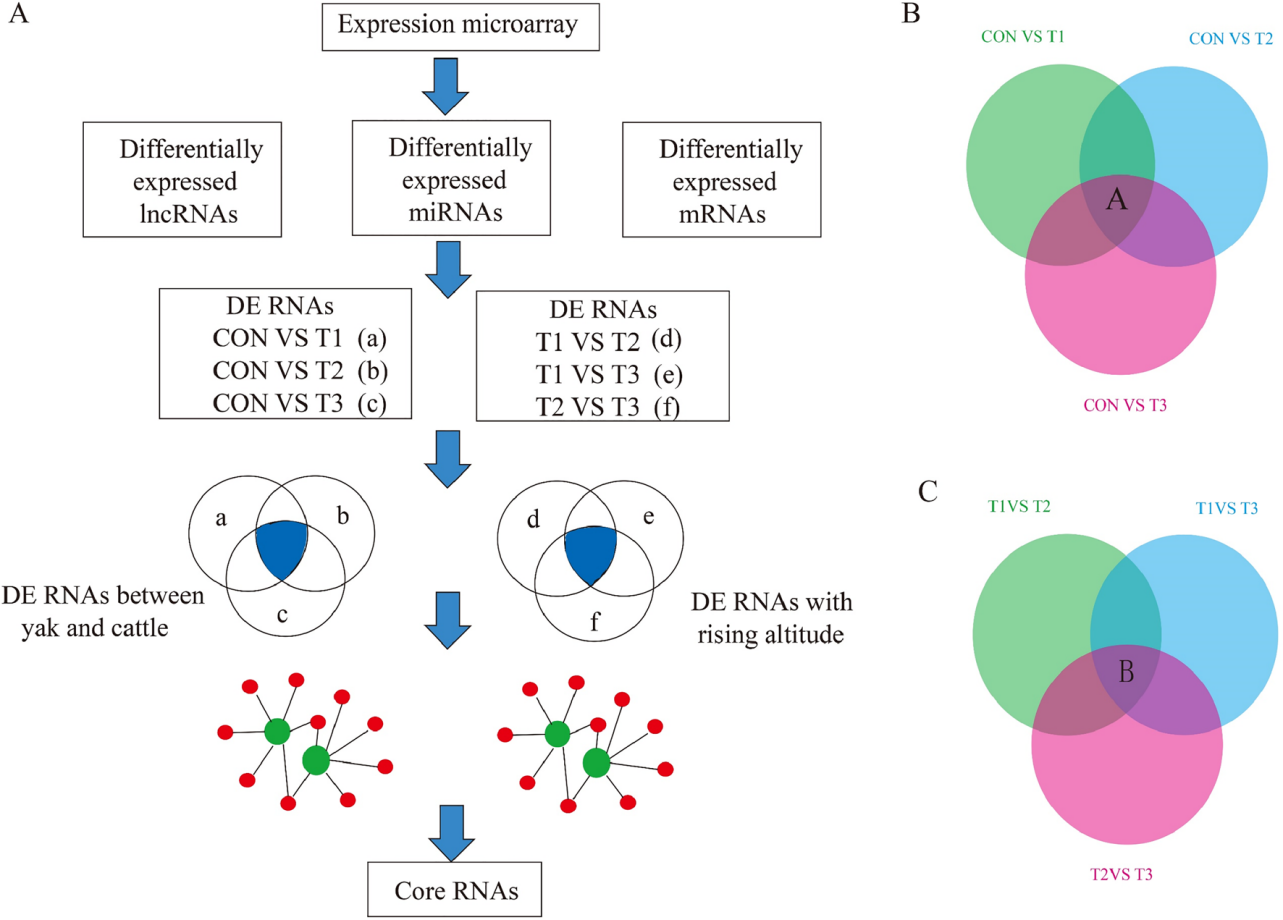
Experimental design and a computational scheme to detect key regulatory molecules and networks in lung tissues from yaks and cattles (**A**). Venn diagram indicated the cattles and yaks intersection (part A) (**B**) and the yaks living at three different altitudes intersection (part B) (**C**).

To identify the key RNAs associated with hypoxic adaptation in yak lung, we analyzed the differentially expressed RNAs in the form of Venn diagrams (Fig. 3). The intersection of the differentially expressed RNAs considered the most interesting candidates, as they represented the main differences in RNA expression. We construct the networks to further interrogate these differentially expressed RNAs which in the intersection of Venn diagrams.

**Figure 4:**
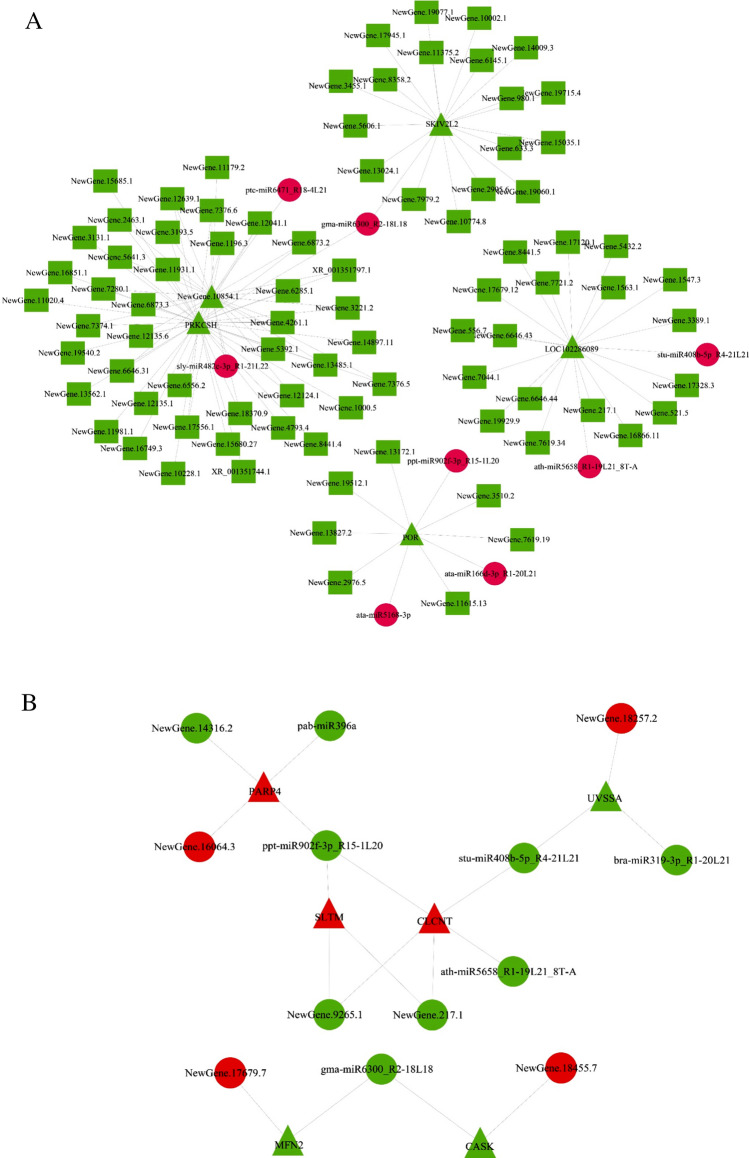
Network of core RNAs in the comparison between yaks and cattles (**A**) and the comparison among yaks living at three different altitudes (**B**). Triangular nodes represent mRNAs; circular nodes represent miRNAs; square nodes represent lncRNAs. Red nodes represent the up-regulated, and the green ones represent the down-regulated transcripts. The network were analyzed using the miRanda software and were visualized by the Cytoscape V3.2 software (http://cytoscape.org/).

The intersection of the differentially expressed RNAs among the CON vs T1, CON vs T2 and CON vs T3 comparisons (Fig. 3B part A) were represented the main differences in RNA expression between yak and cattle. In the networks, all mRNAs and circRNAs were downregulated, and the miRNAs were upregulated. These RNAs, including ptc-miR6471_R18-4L21, gma-miR6300_R2-18L18, sly-miR482e-3p_R1-21L22, ppt-miR902f.-3p_R15-1L20, stu-miR408b-5p_R4-21L21, ath-miR5658_R1-19L21_8T-A, ata-miR166d-3p_R1-20L21 and ata-miR5168-3p, were selected as the most affected RNAs between yak and cattle, and their associated RNAs, including circRNAs and mRNAs (SKIV2L2, PRKCSH, NewGene.10854.1, POR and LOC102286089), identified using miRanda were selected as the core RNAs (Fig. 4A).

The intersection of differentially expressed RNAs identified from the T1 vs T2, T1 vs T3 and T2 vs T3 comparisons represented the differences in RNA expression in yak lung with increasing altitude (Fig. 3C part B). We also constructed networks of the differentially expressed RNAs in the intersection, which included nine downregulated miRNAs (NewGene.14316.2, pab-miR396a, ppt-miR902f.-3p_R15-1L20, stu-miR408b-5p_R4-21L21, bra-miR319-3p_R1-20L21, ath-miR5658_R1-19L21_8T-A, NewGene.9265.1, NewGene.217.1 and gma-miR6300_R2-18L18) and four upregulated miRNAs (NewGene.16064.3, NewGene.18257.2, NewGene.17679.7 and NewGene.18455.7), and their related three upregulated mRNAs (PARP4, SLTM and CLCNT) and three downregulated mRNAs (UVSSA, MFN2 and CASK). There was no related lncRNAs (Fig. 4B).

## Discussion and conclusion

Since the development of next-generation sequencing, a large number of whole-genome sequencing studies of Tibetan mammals have been performed to explore the molecular mechanism of high-altitude adaptation^5,22^. The yak is an ancient species that is unique to the Tibetan Plateau in China. During long-term adaptation to harsh natural environments, such as the hypoxia and low temperatures of the Tibetan Plateau, many genes specifically increase related transcriptional activities and regulate a series of metabolic activities in the body. The lung is a central functional organ in the respiratory system. We compared lung tissue of yaks with cattle to explore the genes associated with hypoxic adaptation. To explore the changes of regulatory mechanisms of genes in the lung tissue of yaks with the increasing of the altitude, we conducted transcriptome analysis of yaks living at three different altitudes in this study.

The transcriptomes of lung tissue in yaks and cattle were examined to identify the major biological processes and mechanisms involved in adaptation to hypoxia. GO analysis revealed that many biological processes were significantly altered. Exposure to hypoxia was mainly associated with the GO terms related to ‘long-chain fatty acid metabolic process’ and ‘protein targeting to peroxisome’. The GO term ‘protein targeting to peroxisome’ is related to peroxisomes, and recent studies have shown that peroxisome proliferator activated receptor (PPAR) expression is detected in the human airway epithelium, bronchiolar submucosa, and airway smooth muscle. This finding will provide new ideas for the treatment of a number of lung-related diseases, such as pulmonary hypertension, acute lung injury, asthma and other common diseases. Therefore, PPAR has important research value for understanding the normal physiological function of the respiratory system and potential application value related to disease prevention and control. Simultaneously, PPARs can regulate the expression of multiple genes at the same time and play important roles in physiological functions such as adipocyte differentiation and regulation of lipid metabolism. Related studies showed that PPARs play an important role in the regulation of energy metabolism by long-chain fatty acids^23^. PPAR-related genes ACOT1 (acyl-coenzyme A thioesterase 1-like) and ACOT2 (acyl-coenzyme A thioesterase 1) were upregulated in this study, which involved in the biosynthesis and metabolism of Fatty Acyl-CoA. ACOT is a key enzyme in the process of fatty acid desaturation and elongation, which regulates the biosynthesis of long-chain fatty acids. Notably, these two genes were also upregulated in another enriched GO term in the comparison between yaks and cattle, ‘long-chain fatty acid metabolic process’. Abca1 (ATP-binding cassette transporter A1) was upregulated in the ‘long-chain fatty acid metabolic process’. Abca1 is a peroxisomal membrane protein that exports cellular cholesterol and phospholipids to apolipoproteins, generating nascent high-density lipoprotein (HDL) particles^24^. Abca1 is highly expressed in adipose tissue and is upregulated during adipogenesis^25,26^ and its expression is critical for regulating adipose tissue cholesterol content^25,27,28^. PPAR is highly expressed in adipocytes and is a major regulator of adipocyte differentiation that controls adipogenesis and lipid metabolism during differentiation^29,30^. The role of PPAR involves transcribing and activating the expression of phosphoenolpyruvate carboxykinase (PEPC) and glycerokinase (GK) and participating in the production of triacylglycerol through different pathways. Under the action of lipoprotein lipase (LPL), PPAR adjusts the glucose content and then affects the final synthesis of triglycerides by affecting 3-phosphoglycerate (Figure S5). Therefore, PPAR plays an important role in the final synthesis of triglycerides. It is well known that there are two main types of adipose tissue, namely, white adipose tissue (WAT) and brown adipose tissue (BAT)^30^. WAT can store adipose tissue and is a very active tissue^31^, and BAT can burn (white) adipose tissue in the body via a heat-generating function that uncouples protein resulting in oxidation of lipid in mitochondria. BAT is essential for animals under cold plateau conditions, which is a hot area of research on the plateau animals adapt to the cold environment in the plateau area. PPAR is a key regulator of BAT cell differentiation and heat production^32^. The PPAR signaling pathway can promote the differentiation of mesenchymal progenitor cells into BAT cells, helping to produce heat and increase energy consumption. And there were also studies have shown that BAT expresses heat-producing markers that include PPAR^33^. PPAR helps animals resist the cold environment of the plateau by regulating BAT differentiation and heat production and increasing nonshivering thermogenesis to maintain a constant body temperature^34^. We infer that yaks maybe increase BAT cell differentiation and heat production by upregulated PPAR to resist the cold environment of the plateau.

**Figure 5:**
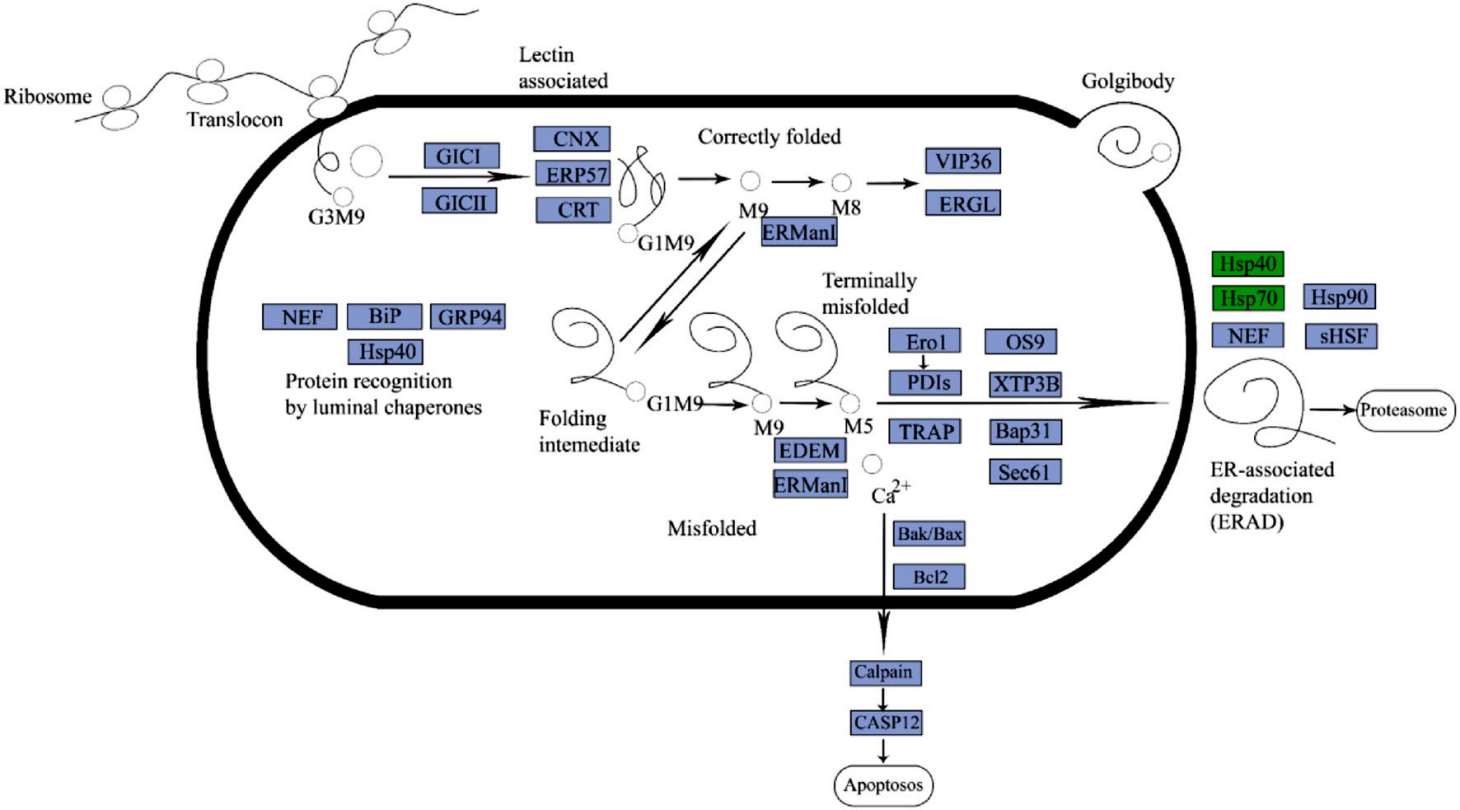
Protein processing in the endoplasmic reticulum at high altitudes (The purple boxes represent enzymes or genes, green box represent down-regulated). The pathway was visualized by the Adobe Illustrator software (https://www.adobe.com/cn/products/illustrator.html).

The most significantly enriched pathway in the lungs of yaks compared to those of cattle in response to hypoxia was ‘protein processing in the endoplasmic reticulum (ER)’. Disruption at any stage of the protein process may affect the function of the protein and even impact the normal function of the whole cell due to animals suffer from the effects of cold and hypoxia in plateau environments. It is hypothesize that the protein-folding mechanisms would increase accordingly, as high altitudes may impact protein processing. In the ER, proteins are subjected to strict quality control (QC) to ensure correct folding or to facilitate degradation of misfolded polypeptides through a series of tightly regulated processes known as ER-associated degradation (ERAD). The QC system consists of calreticulin (CRT) and lectin chaperones calnexin (CNX), as well as the cochaperone ERp57, a glycoprotein-specific thiol-disulfide oxidoreductase; ‘calreticulin/calnexin cycle’ is composed of these factors (Fig. 5)^35^. In this study, the expression of protein disulfide isomerase A4 (PDIA4) was upregulated to promote correct protein folding. PDI may interact to recover part of the function of CNX, thus promoting the recovery and maintenance of normal lung function in yaks under high-altitude conditions^36^. The misfolded proteins in the ER lumen are recognized by luminal chaperones such as HSP40, NEF, GRP94 and BIP, which transfer misfolded proteins to the ER membrane for ubiquitin-dependent degradation (Fig. 5)^37^. Since the accumulation of misfolded proteins hinders ER function and can ultimately lead to cell death, rapid removal of misfolded polypeptides through the ERAD pathway is a critical process. ERAD is involved in chaperones of the HSP family that act synergically with ubiquitin ligases^38^. The HSP family is a well-known gene family that responds to heat stress. In response to low temperature, the expression levels of HSP70 and HSP40, which participate in the ERAD process, were downregulated (Fig. 5), which is likely to promote the degradation of misfolded proteins and to maintain cell homeostasis. The hypoxia-induced gene HYOU1 was also upregulated in the ‘protein processing in the ER’ pathway, indicating that hypoxia tolerance and temperature tolerance are associated with each other^39^ and suggesting that yaks adapt to cold and hypoxia simultaneously. BAX and CAPN1, which are involved in apoptosis-related physiological processes, are upregulated by hypoxia in the ‘protein processing in the ER’ pathway. Apoptosis and programmed cell death are critical for the removal of dead cells and are linked to various biological processes. Defects in apoptosis may lead to cancer and autoimmune diseases^40^. The upregulation of BAX and CAPN1 expression in yaks is involved in adaptation to hypoxia. In the horrible conditions of Tibetan plateau, yaks increase protein-folding rates to maintain the function of whole cells.

In a comparison of yaks living at three different altitudes to identify which processes were significantly enriched with increasing altitude, the most overrepresented biological function in the lung in response to environmental stress was ‘immune response’, including ‘regulation of lymphocyte migration’ ‘immunoglobulin production’ ‘complement activation’ ‘regulation of complement activation’ ‘Fc-gamma receptor signaling pathway involved in phagocytosis’ and ‘leukocyte migration’. The overall atmospheric oxygen content of the Tibetan Plateau is less than 60% of that of the plain. In the state of hypoxia, the functional damage to the body caused by insufficient oxygen supply or impaired oxygen use affects normal life activities, the permeability of the yak lung tissue is increased, and the expression of various inflammatory mediators and their receptors is significantly increased. Previous studies found that many genes were related to DNA repair, blood circulation, and immunity in Tibetan pigs compared with local Chinese pig breeds^41^. Yaks can live in an extremely hypoxic environment for a long time without any hypoxic injury to the lungs. The significant accumulation of the complement cascade in the lungs of yaks is a good illustration of how yaks living in a hypoxic environment have developed molecular mechanisms of resistance to avoid damage caused by hypoxia to respiratory organs such as the lungs.

**Figure 6:**
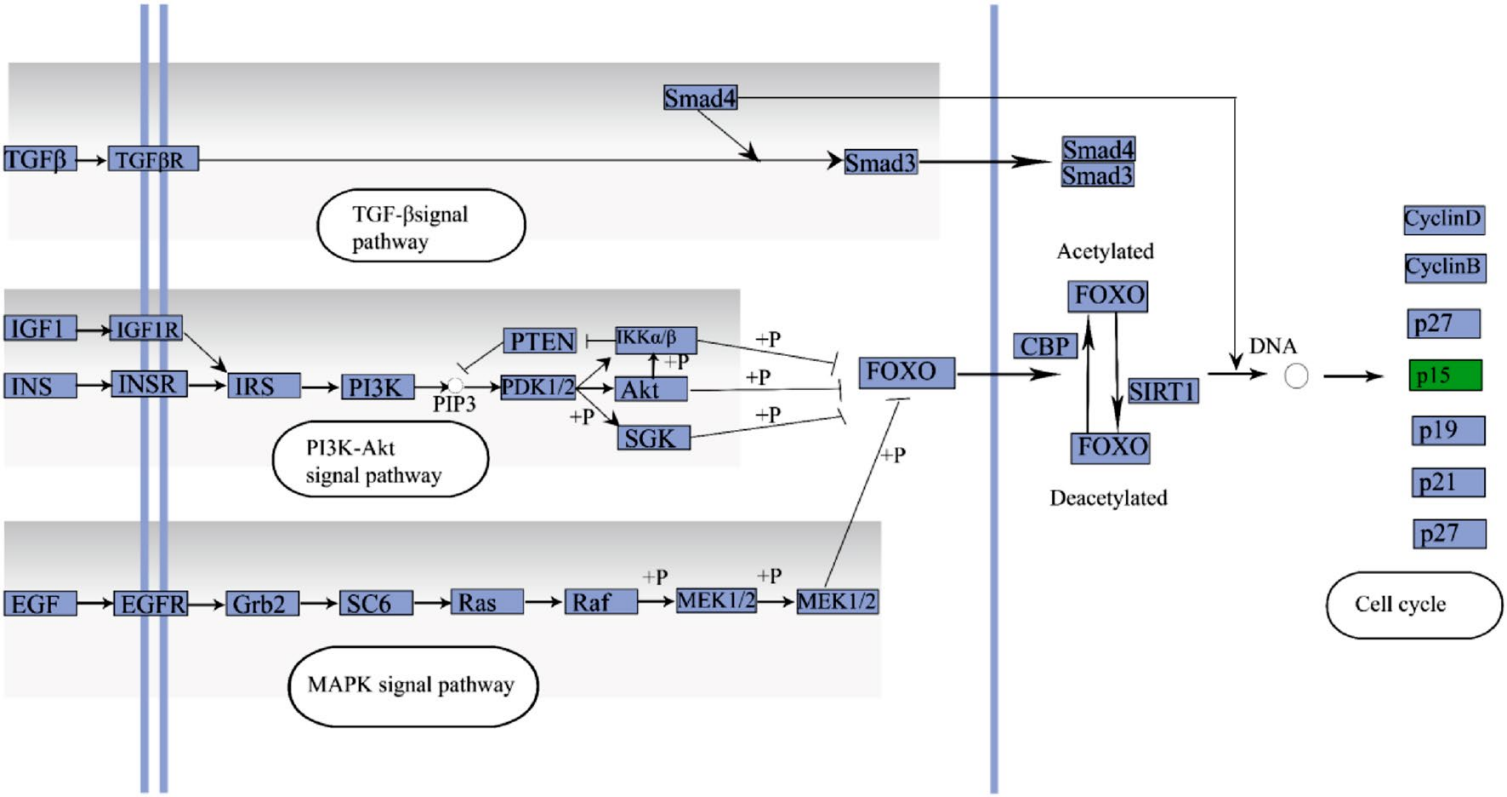
Cell cycle at high altitudes (The purple boxes represent enzymes or genes, green box represent down-regulated). The pathway was visualized by the Adobe Illustrator software (https://www.adobe.com/cn/products/illustrator.html).

The FOXO signaling pathway was also enriched in a comparison of yaks living at three different altitudes. The FOXO signaling pathway can regulate the expression of the cell cycle and apoptosis-related genes to inhibit the cell cycle and promote apoptosis, thereby inhibiting cell proliferation^42^. The phosphoinositide-3-kinase (PI3K)/protein kinase B (Akt) signaling pathway, mitogen-activated protein kinase (MAPK) signaling pathway and transforming growth factor-beta (TGF-beta) pathway are the three main upstream pathways of FOXO (Fig. 6). The PI3K/AKT signaling pathway plays an extremely important role in FOXO regulation. Akt is the first kinase that has been shown to inhibit FOXO function. The MAPK signaling pathway is another important proliferative and antiapoptotic pathway upstream of FOXO. Its activation can affect the activity of effector molecules such as downstream cell cycle regulators and apoptosis-related proteins, leading to uncontrolled cell cycle and apoptosis abnormalities that play an important role in the occurrence, development, invasion and metastasis of tumors^43,44^. The MAPK pathway and PI3K pathway are two important pathways for transmitting cell proliferation signals, and these pathways are known to be related to adaptation to hypoxia^18,45^. There is a certain degree of interaction between these two pathways. The FOXO protein is at the intersection of these two signaling pathways and activates the activity of FOXO transcription factors involved in cell proliferation and apoptosis regulation^46^. After stimulation by cell proliferation signals including growth factors and other factors^47^, these two signaling pathways are activated, conserved sites in FOXO protein molecules are phosphorylated, this leads to the loss of transcriptional activity and the loss of regulatory effects on the cell cycle and apoptosis, thereby promoting cell proliferation^48,49^ and influencing the cell cycle and apoptotic events via the regulation of downstream cell cycle and apoptosis-related signaling molecules^50–53^. Transforming growth factor-beta (TGF-beta) is a multifunctional regulatory peptide family that can control a variety of cell biological functions, including cell proliferation, differentiation, metastasis, apoptosis, adhesion and angiogenesis. TGF regulates the expression of many cell cycle regulators, such as the cytostatic factor p15, p19 and p21, by activating Smad transcription factors to make it bind to the DNA-binding domains of FOXO1, FOXO3a, and FOXO4^54,55^. Under hypoxic conditions, cells are exposed to environmental stresses, and apoptosis is induced by hyperpermeability of the inner mitochondrial membrane, the generation of reactive oxygen species (ROS) or the activation of stress-activated protein kinase (SAPK)^56^. Yaks regulate FOXO through the PI3K-AKT, MAPK and TGF-beta pathways and downregulate P15 to promote cell survival and proliferation, preventing cell apoptosis under low-oxygen stress at high altitudes (Fig. 6). Our observations related to the FOXO pathway highlight the potential molecular mechanism used to overcome hypobaric hypoxia. It has been widely reported that activating PI3K/AKT can protect cells from apoptosis in rats^57^ and multiple cancers^58^. Yaks have used the same mechanism to respond to hypoxic stress. Unlike those of malignant tumors, the growth and division of yak cells are still under control, which may provide insights for biomedical research on treatments for cancer.

After detecting abundant differentially expressed RNAs between yak and cattle, we constructed a computational scheme (Fig. 3) to identify the key regulatory molecules and networks involved in hypoxic adaptation. Here, we found many novel RNAs to be candidates for hypoxic adaptation compared with previous studies that performed microarray analyses. First, the overlap RNAs in Venn diagrams showed the intersection (part A and part B in Fig. 3) according to the experimental design. Differentially expressed RNAs in part A were majorly associated with difference between yak and cattle whereas RNAs in part B were associated with difference among yaks living at three different altitudes. Second, although the mRNA-miRNA-lncRNA interaction networks included predictable relationship between mRNA, miRNA and lncRNA according to the current database and bioinformatics analysis, the regulatory networks provided important information regarding core hypoxic adaptation-associated RNAs. Third, the core mRNAs in part A (differences of yak and cattle) predominantly encoded protein processing in endoplasmic reticulum and heme binding, such as PRKCSH and POR. Conversely, the core mRNA-encoded proteins in part B (differences of yaks living at three different altitudes) were mainly enriched in apoptosis, p53 signaling pathway, DNA repair and immunity, such as PARP4, UVSSA and MFN2. The function of core mRNAs in two intersectional parts was also confirmed with GO and KEGG analysis mentioned above, respectively.

Our study has many advantages over previous microarray about hypoxic adaptation in lung tissue of yak. Instead of focusing on single genes or a group of candidate genes previously demonstrated to be associated with hypoxic adaptation, the high-throughput sequencing method provided complete coverage and greater dynamic range of the transcriptome, and allowed an unbiased evaluation of the biological processes underlying hypoxic adaptation in lung tissue of yak. Finally, generating network interactions of coding and ncRNA enabled us to elucidate the functional complexity of the yak transcriptome and identify key regulatory molecules and networks.

## Methods

### Ethics statement

The procedures for animal care were approved by the Gansu Agricultural University Animal Care and Use Committee of the Gansu Agricultural University, and all experiments were conducted in accordance with approved relevant guidelines and regulations. The study was carried out in compliance with the ARRIVE guidelines (http://www.nc3rs.org.uk/page.asp?id=1357).

### Sample collection and transcriptome sequencing

We collected lung tissue samples from 9 indigenous adult male yaks with a gross body weight of 200 kg and an age of 4–6 years old from three different altitudes (3400 m, 4200 m and 5000 m), with three repetitions per altitude, and two indigenous adult male cattle with a gross body weight of 400 kg and an age of 4–6 years old from 1500 m as a low-altitude control. These samples included three yaks from Maqu County in the Gannan Tibetan Autonomous Prefecture of Gansu Province representing an altitude of 3400 m, three yaks from Dangxiong County, Linzhou County and Bange County representing an altitude of 4200 m, three yaks from Anduo County in the Tibetan Autonomous Region representing an altitude of 5000 m, and two indigenous Zaosheng cattle from Ningxian County in Gansu Province, as the T1, T2, T3 and CON groups, respectively (Table 2, Figure S6). Animals of breeding age are reported to have relatively stable gene expression, so we chose animals of this age. Before collecting samples, we performed a physical examination of the animals to ensure the health of the animals. All tissues were immediately frozen in liquid nitrogen until RNA extraction. Total RNA was extracted using TRIzol reagent, and the purity of the isolated RNA was determined by agarose gel electrophoresis. Libraries were prepared from the resulting total RNA and sequenced on the Illumina HiSeq 2500 platform with paired-end 150 bp reads. For each samples, 10G of data were generated.

### Transcriptome assembly

RNA-seq reads were preprocessed, and low-quality areas that affected the data quality were removed for subsequent analysis. The raw data were filtered under a series of steps as follow. For RNA data, The BWA algorithm was used to remove the low quality reads with a mass threshold of 30. Then joint sequence, unknown base calls (N) and sequences of length less than 60 bp were trimmed out from RNA raw data. For the raw data of miRNA, unknown base calls (N), reads without 3′ adapters, low quality reads (> 15% of bases whose Phred scores were ≤ 20), reads containing poly-A/T or the length was not within the required range (15-34nt) were filtered to generate clean data. The clean reads were mapped to the reference genome ftp://ftp.ncbi.nlm.nih.gov/genomes/refseq/vertebrate_mammalian/Bos_mutus/annotation_releases/current/GCF_000298355.1_BosGru_v2.0/GCF_000298355.1_BosGru_v2.0_genomic.fna.gz using HISAT2 (v2.0.5) (http://ccb.jhu.edu/sofware/hisat2/index.shtml). The resulted bam files of each sample were feed to StringTie (v1.3.2d) to obtain transcript information of each sample, then merged all transcript to get results of assembly.

### Identification of lncRNA and miRNA

Potential lncRNAs were identified from the assembled transcripts through the following highly stringent criterion: (1) transcript length is not less than 200 nt; (2) transcript expression is more than 3 reads; (3) transcripts with reads coverage of less than 5 were eliminated; (4) compared with the annotation file of the species to screen the known mRNA and other non-coding RNA (rRNA, tRNA, snoRNA and snRNA) according to the gffcompare software; (5) according to the information of class code (“u”, “i”, “x”), the potential lincRNA, intronic lncRNA and anti-sense lncRNA were screened; (6) the calculation of the protein-coding potency of transcripts was performed using four software programs: CNCI (Coding-Non-Coding-Index) (score < 0), CPC (Coding Potential Calculator) (score < 0), Pfam-scan (E-value < 0.001), and PhyloCSF. Predicted transcripts with coding potential in any or all of the four tools were filtered out.

For miRNA, unique reads of Quality Control first aligned to the Rfam RNA^59^ families database to filter other small RNA species. BLAST parameters: (1) E value no more than 1; (2) Number of mismatched bases no more than 1; (3) Score ≥ 15 (At least 15 bases). Compare the mature miRNA sequences of this species in the miRBase database^60^.Those that could not be compared were then compared with the mature miRNA sequences of other species in the miRBase database to identify known miRNA from sequencing data. For sequences not identified as known miRNA, bowtie2^61^ was first used to locate them on the reference genome. Then MIREAP was used to predict the novel miRNA based on the results of localization.

### Qualification and differential analysis of RNAs expression level

Previously, read counts of each sample were obtained, for mRNA and lncRNA, FPKM (Fragments Per Kilobase of transcript per Million fragments mapped) of each samples were obtained, for miRNA, RPM (reads per million) values were calculated according to total counts of each sample. Then completed the analysis of differentially expressed RNAs (mRNA, lncRNA and miRNA)between CON vs T1, CON vs T2, CON vs T3, T1 vs T2, T1 vs T3, T2 vs T3 by DESeq2 (http://www.bioconductor.org/packages/release/bioc/html/DESeq.html), considering calculated P value accomplished multiple hypothesis testing (Benjamini Y, Hochberg Y 1995. Controlling the false discovery rate: a practical and powerful approach to multiple testing. J R Stat Soc B 57: 12. ), therefore, an adjusted P value < 0.05 and |log2foldchange|> 1 was applied to detect differential expression RNAs^62^.

### Target gene prediction of lncRNAs and miRNAs

For lncRNAs, mRNAs with a high Spearman correlation coefficient (P ≥ 0.9) were selected as the trans-targets. mRNAs with distances less than 50 kb were selected as cis-targets. For miRNAs, miRanda^63^ and RNAhybrid^64^ was used to predict targets of known or novel miRNAs. The principle of miRanda prediction was based on seed region sequence alignment. The results were filtered by the parameters -sc 160 -en -20.

### Enrichment analysis

For the analysis of differentially expressed RNAs (mRNA, miRNA, lncRNA), we applied clusterProfiler (Guangchuang Yu guangchuangyu@gmail.com ) Packag in R to identity gene ontology (GO) and KEGG pathway categories, a cutoff value of 0.05 from the chi-square test to filter significantly enriched GO and KEGG pathway categories^65,66^. All tests completed multiple hypothesis testing according to Benjamini–Hochberg approach (Benjamini Y, Hochberg Y 1995. Controlling the false discovery rate: a practical and powerful approach to multiple testing. J R Stat Soc B 57: 12).

### Quantitative real-time (RT)-qPCR analysis

RT-qPCR was performed on 7 mRNAs and 5 lncRNAs elected from the RNAs sequencing data according to potential functional importance. Primers were designed according to sequencing data from the lung transcriptome of yaks using Primer Premier 5.0. PCR amplification and specificity were examined via melting curve analysis. The relative expression of target gene transcripts was calculated using the comparative Ct method, and SPSS software was used for statistical analysis.

## Supplementary Information

Supplementary Information 1.

Supplementary Information 2.

## Data Availability

Sequence data have been deposited with the GenBank Data Libraries under Accession GSE153956 and GSE153962.
