# Supplementary material for: Molecular mechanisms detected in yak lung tissue via transcriptome-wide analysis provide insights into adaptation to high altitudes
Source: Sci Rep. 2021 Apr 8;11:7786. doi: 10.1038/s41598-021-87420-7 (PMC8032655; doi:10.1038/s41598-021-87420-7)
Supplement: Supplementary file 1 — Supplementary Information 1. [file 41598_2021_87420_MOESM1_ESM.docx]

**Molecular mechanisms detected in yak lung tissue via transcriptome-wide analysis provide insights into adaptation to high altitudes**

Qianyun Ge^a^, Yongbo Guo^b^, Wangshan Zheng^b^, Shengguo Zhao^a,^* Yuan Cai^a,^*, Xuebin Qi ^b,^*

* Corresponding author:

Shengguo Zhao, E-mail: zhaosg@gsau.edu.cn

Yuan Cai, E-mail: caiyuan@gsau.edu.cn

Xuebin Qi, E-mail: qixuebin@mail.kiz.ac.cn

^a^ College of Animal Science and Technology, Gansu Agricultural University, Lanzhou 730070, China

^b^ State Key Laboratory of Genetic Resources and Evolution, Kunming Institute of Zoology, Chinese Academy of Sciences, Kunming 650223, China


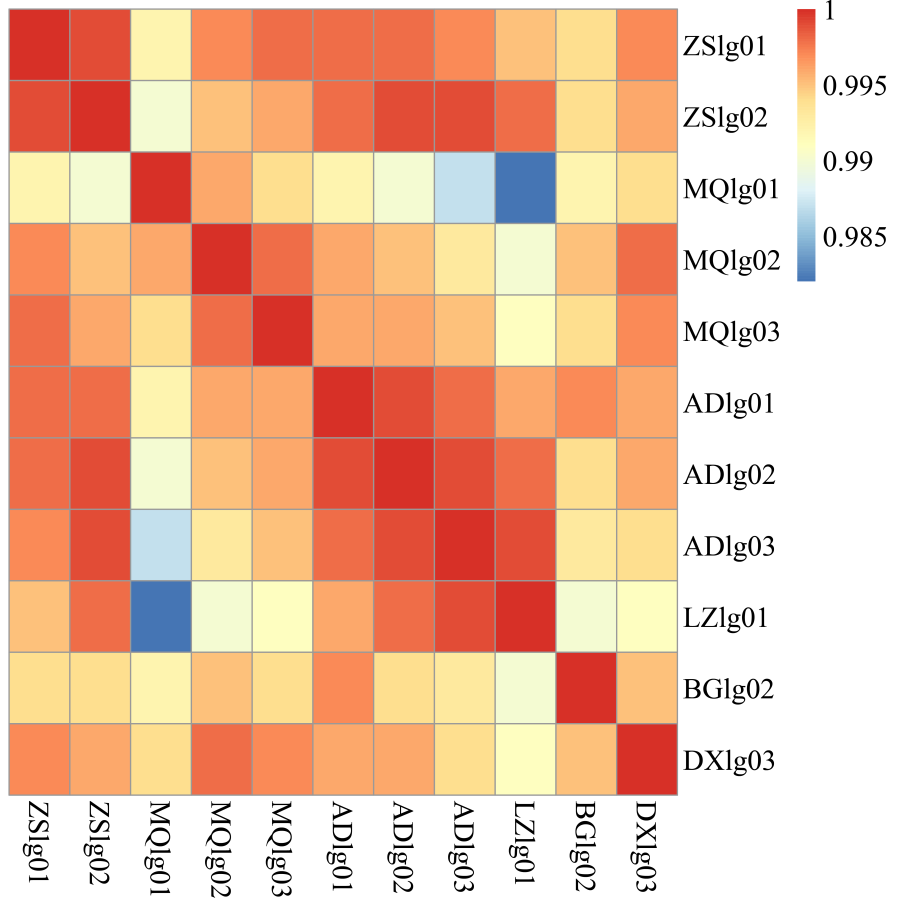


Figure S1. The clustering of samples based on the variations in the gene expression.


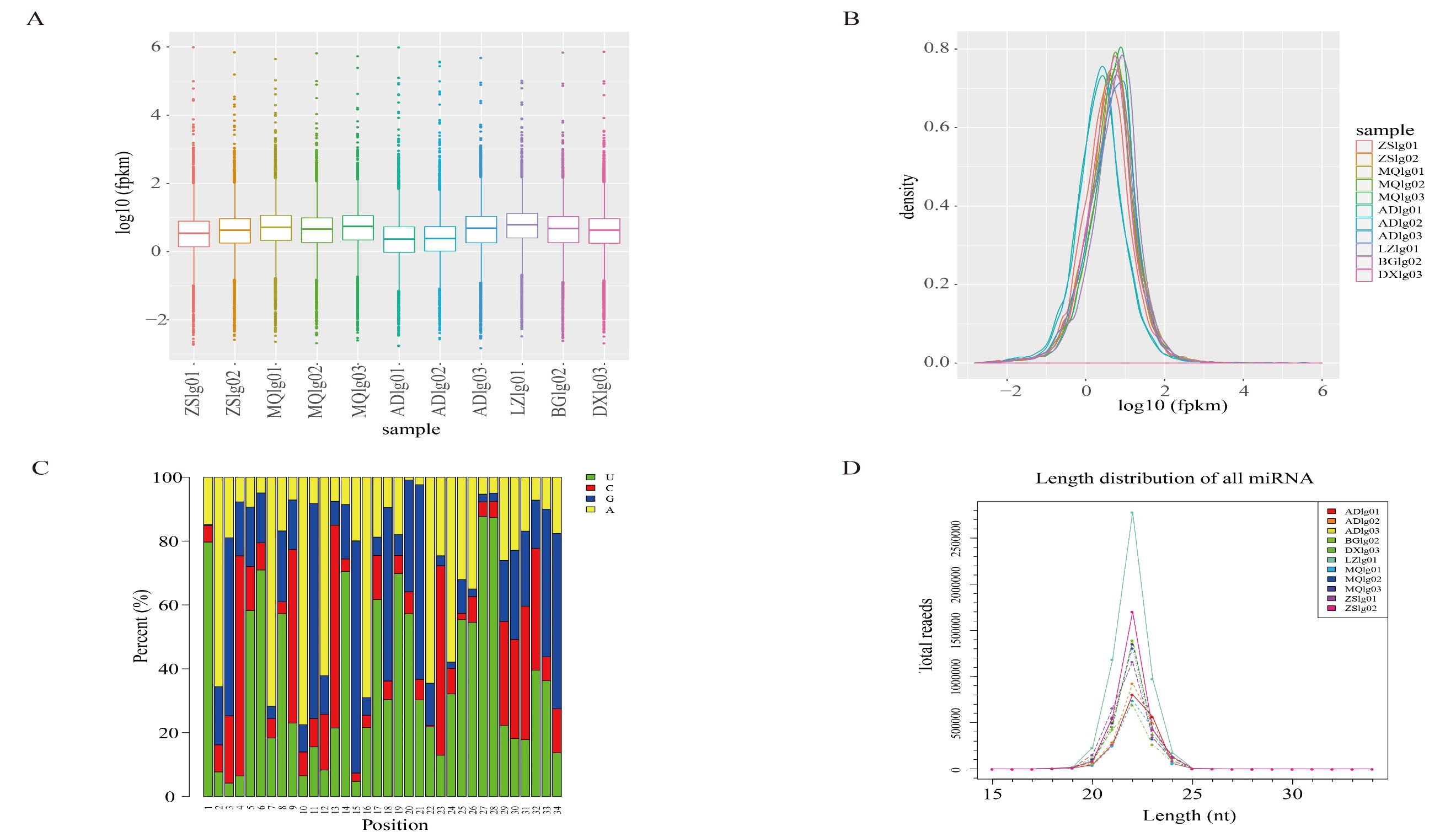


Figure S2. The expression pattern of lncRNAs and miRNAs detected by high-throughput RNA sequencing. Box plots of transcripts per million clean tags (TPM) value (A) and density distribution (B) for lncRNAs in all sampes. Base distribution (C) and length (D) of miRNAs in all samples.


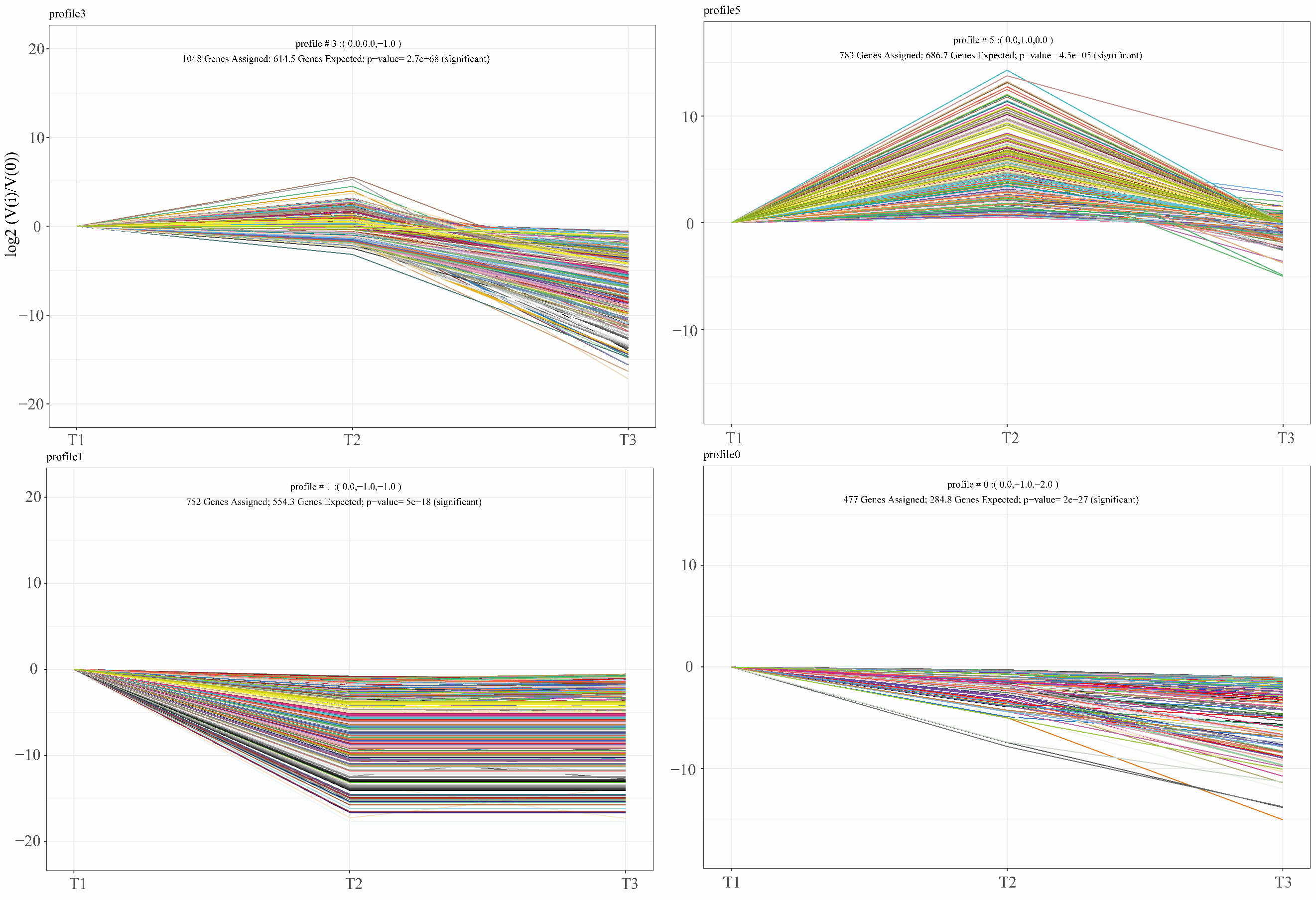


Figure S3. Profiles of altitude-dependent differentially expressed genes in yaks at three different altitudes. The modules indicate different expression patterns that were found to be significantly enriched via a STEM analysis (nonsignificant modules are not shown). STEM analysis was performed using the OmicShare tools, a free online platform for data analysis (http://www.omicshare.com/tools).


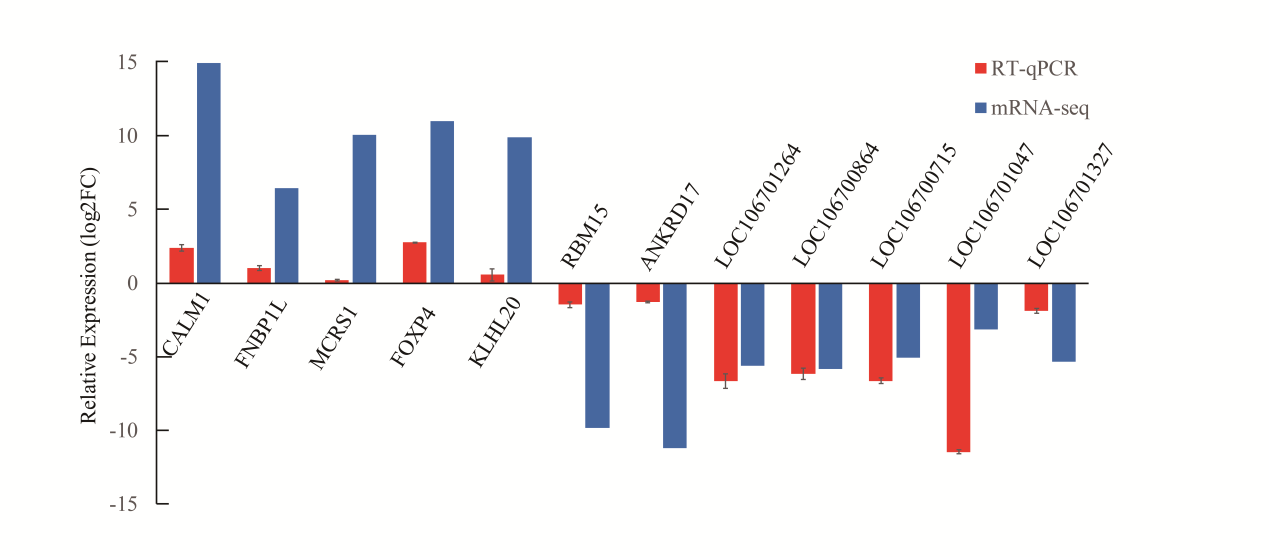


Figure S4. Comparison of mRNA and lncRNA expression levels determined by the RNA-seq and RT-qPCR methods. The relative expression values were normalized to β-actin gene expression. The red histograms represent relative expression values determined by RT-qPCR; the blue histograms represent the FPKM values determined by RNA-seq.


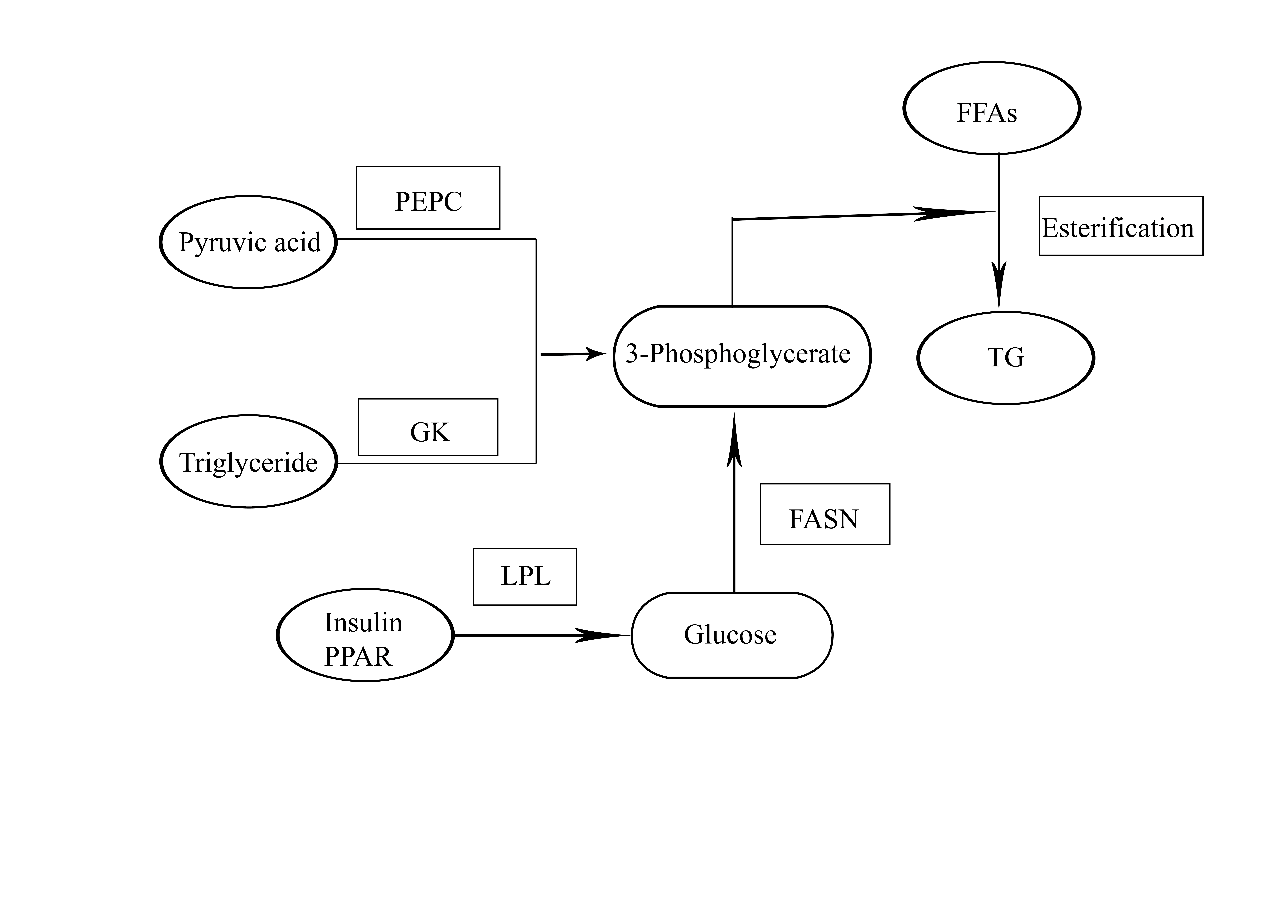


Figure S5. Effects of PPAR during triacylglycerol production. TG: triglycerides; FFAs: free fatty acids; PEPC: phosphoenolpyruvate carboxykinase; GK: glycerokinase; LPL: lipoprotein lipase; FASN: Fatty acid synthase.


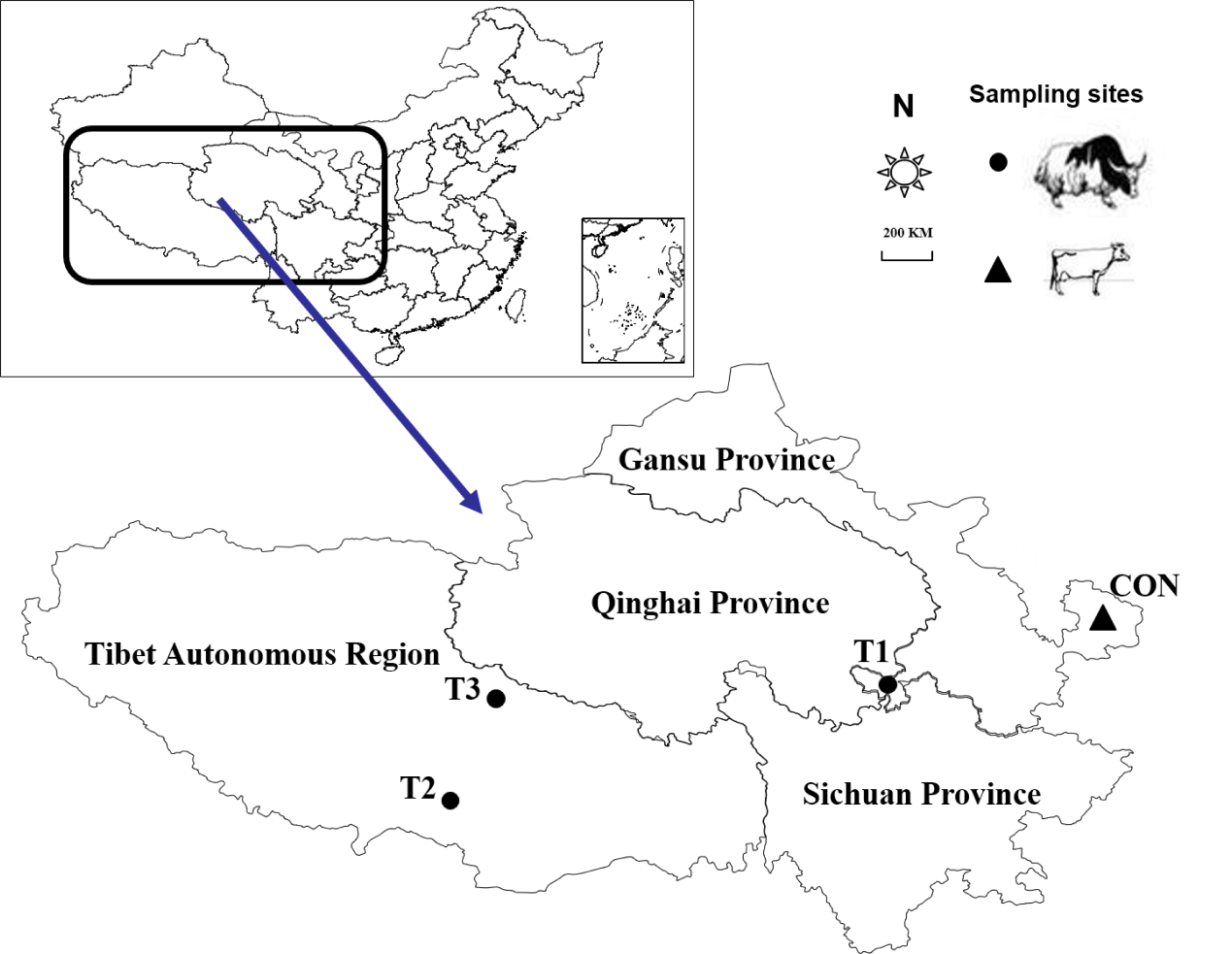


Figure S6. The map shows sampling location distribution and was visualized by the Adobe Illustrator software (https://www.adobe.com/cn/products/illustrator.html).
