# Supplementary material for: Molecular mechanisms detected in yak lung tissue via transcriptome-wide analysis provide insights into adaptation to high altitudes
Source: Sci Rep. 2021 Apr 8;11:7786. doi: 10.1038/s41598-021-87420-7 (PMC8032655; doi:10.1038/s41598-021-87420-7)
Supplement: Supplementary file 2 — Supplementary Information 2. [file 41598_2021_87420_MOESM2_ESM.docx]

**Molecular mechanisms detected in yak lung tissue via transcriptome-wide analysis provide insights into adaptation to high altitudes**

Qianyun Ge^a^, Yongbo Guo^b^, Wangshan Zheng^b^, Shengguo Zhao^a,^* Yuan Cai^a,^*, Xuebin Qi ^b,^*

* Corresponding author:

Shengguo Zhao, E-mail: zhaosg@gsau.edu.cn

Yuan Cai, E-mail: caiyuan@gsau.edu.cn

Xuebin Qi, E-mail: qixuebin@mail.kiz.ac.cn

^a^ College of Animal Science and Technology, Gansu Agricultural University, Lanzhou 730070, China

^b^ State Key Laboratory of Genetic Resources and Evolution, Kunming Institute of Zoology, Chinese Academy of Sciences, Kunming 650223, China

**Supplementary materials**

Table S1. Summary of sequencing data quality and statistics of the transcriptome assemblies.

| Sample | Duplicates | Raw reads (M) | Clean reads (M) | Clean bases (G) | Q20 (%) | Q30 (%) | Total mapped | Uniquely mapped |
| --- | --- | --- | --- | --- | --- | --- | --- | --- |
| CON | ZSlg01 | 74.864 | 73.483 | 10.958(97.6%) | 10.759(98.2%) | 10.337(94.3%) | 65.162 | 65.162(88.68%) |
|  | ZSlg02 | 78.358 | 76.714 | 11.422(97.2%) | 11.079(97.0%) | 10.485(91.8%) | 66.666 | 66.666(86.90%) |
| T1 | MQlg01 | 93.487 | 92.431 | 13.782(98.3%) | 13.570(98.5%) | 13.091(95.0%) | 80.296 | 80.296(86.87%) |
|  | MQlg02 | 91.873 | 91 | 13.587(98.6%) | 13.408(98.7%) | 12.997(95.7%) | 82.703 | 82.703(90.88%) |
|  | MQlg03 | 69.43 | 68.33 | 10.197(97.9%) | 10.018(98.2%) | 9.639(94.5%) | 62.38 | 62.380(91.29%) |
| T2 | LZlg01 | 82.151 | 81.188 | 12.098(98.2%) | 11.907(98.4%) | 11.485(94.9%) | 72.621 | 72.621(89.45%) |
|  | BGlg02 | 71.79 | 70.376 | 10.476(97.3%) | 10.161(97.0%) | 9.611(91.7%) | 64.234 | 64.234(91.27%) |
|  | DXlg03 | 80.375 | 79.419 | 11.826(98.1%) | 11.633(98.4%) | 11.208(94.8%) | 71.896 | 71.896(90.53%) |
| T3 | ADlg01 | 88.463 | 87.25 | 12.995(97.9%) | 12.788(98.4%) | 12.335(94.9%) | 68.698 | 68.698(78.74%) |
|  | ADlg02 | 87.835 | 85.665 | 12.739(96.7%) | 12.531(98.4%) | 12.098(95.0%) | 53.963 | 53.963(62.99%) |
|  | ADlg03 | 113.71 | 112.659 | 16.789(98.4%) | 16.583(98.8%) | 16.107(95.9%) | 103.072 | 103.072(91.49%) |

ZSlg01 and ZSlg02 indicate libraries derived from the lung tissue of Zaosheng cattle in two biological replicates.

MQlg01, MQlg02, and MQlg03 indicate libraries derived from the lung tissue of yaks living at an altitude 3,400 m in three biological replicates.

LZlg01, BGlg02, and DXlg03 indicate libraries derived from the lung tissue of yaks living at an altitude 4,200 m in three biological replicates.

ADlg01, ADlg02, and ADlg03 indicate libraries derived from the lung tissue of yaks living at an altitude 5,000 m in three biological replicates.

These designations are used similarly hereafter.

Table S2. Summary of sequencing data quality and statistics of the miRNA data.

| Sample | Duplicates | Raw Reads(M) | Raw Bases(G) | Raw Q20(G) | Raw Q30(G) | Clean Reads(M) | Clean Bases(G) | Clean Q20(G) | Clean Q30(G) | Average Length(bp) |
| --- | --- | --- | --- | --- | --- | --- | --- | --- | --- | --- |
| CON | ZSlg01 | 8.908 | 1.336 | 0.931(69.7%) | 0.810(60.6%) | 8.226(92.3%) | 0.180(13.4%) | 0.179(99.8%) | 0.178(99.1%) | 21.8 |
|  | ZSlg02 | 9.282 | 1.402 | 1.114(79.5%) | 0.963(68.7%) | 8.260(89.0%) | 0.182(13.0%) | 0.181(99.9%) | 0.181(99.7%) | 22 |
| T1 | MQlg01 | 9.283 | 1.402 | 1.123(80.1%) | 0.981(70.0%) | 9.066(97.7%) | 0.197(14.1%) | 0.197(99.9%) | 0.197(99.8%) | 21.8 |
|  | MQlg02 | 10.633 | 1.606 | 1.253(78.1%) | 1.085(67.6%) | 8.646(81.3%) | 0.189(11.8%) | 0.189(99.9%) | 0.188(99.7%) | 21.8 |
|  | MQlg03 | 9.917 | 1.497 | 1.190(79.5%) | 1.032(68.9%) | 8.899(89.7%) | 0.196(13.1%) | 0.196(99.9%) | 0.195(99.6%) | 22 |
| T2 | LZlg01 | 13.171 | 1.989 | 1.591(80.0%) | 1.380(69.4%) | 12.474(94.7%) | 0.271(13.6%) | 0.270(99.9%) | 0.269(99.6%) | 21.7 |
|  | BGlg02 | 6.713 | 1.007 | 0.704(69.9%) | 0.615(61.1%) | 6.226(92.7%) | 0.138(13.7%) | 0.138(99.8%) | 0.137(99.2%) | 22.2 |
|  | DXlg03 | 10.239 | 1.546 | 1.223(79.1%) | 1.059(68.5%) | 9.069(88.6%) | 0.199(12.9%) | 0.199(99.9%) | 0.199(99.7%) | 22 |
| T3 | ADlg01 | 11.611 | 1.753 | 1.407(80.2%) | 1.221(69.6%) | 11.315(97.4%) | 0.246(14.0%) | 0.246(99.9%) | 0.245(99.6%) | 21.7 |
|  | ADlg02 | 10.935 | 1.651 | 1.324(80.2%) | 1.157(70.0%) | 10.633(97.2%) | 0.233(14.1%) | 0.232(99.9%) | 0.232(99.7%) | 21.9 |
|  | ADlg03 | 9.257 | 1.398 | 1.098(78.5%) | 0.953(68.2%) | 7.850(84.8%) | 0.172(12.3%) | 0.172(99.9%) | 0.171(99.7%) | 21.9 |

Table S3. Distribution of known miRNAs and novel miRNAs in each sample.

| miRNA | ZSlg01 | ZSlg02 | MQlg01 | MQlg02 | MQlg03 | LZlg01 | BGlg02 | DXlg03 | ADlg01 | ADlg02 | ADlg03 |
| --- | --- | --- | --- | --- | --- | --- | --- | --- | --- | --- | --- |
| known_miRNA | 29 | 107 | 92 | 112 | 111 | 88 | 20 | 117 | 110 | 107 | 107 |
| novel_miRNA | 334 | 303 | 292 | 335 | 331 | 409 | 341 | 260 | 258 | 281 | 303 |

Table S4. Targeted genes of miRNA.

| Group1 | CON | CON | CON | CON | CON | CON | T1 | T1 | T1 | T1 | T2 | T2 |
| --- | --- | --- | --- | --- | --- | --- | --- | --- | --- | --- | --- | --- |
| Group2 | T1 | T1 | T2 | T2 | T3 | T3 | T2 | T2 | T3 | T3 | T3 | T3 |
| Regulate | UP | DOWN | UP | DOWN | UP | DOWN | UP | DOWN | UP | DOWN | UP | DOWN |
| TargetGene | 1363 | 1695 | 519 | 278 | 1192 | 2274 | 947 | 634 | 951 | 551 | 1674 | 1205 |

Table S5. Targeted genes of lncRNA.

| Group1 | CON | CON | CON | CON | CON | CON | T1 | T1 | T1 | T1 | T2 | T2 |
| --- | --- | --- | --- | --- | --- | --- | --- | --- | --- | --- | --- | --- |
| Group2 | T1 | T1 | T2 | T2 | T3 | T3 | T2 | T2 | T3 | T3 | T3 | T3 |
| Regulate | UP | DOWN | UP | DOWN | UP | DOWN | UP | DOWN | UP | DOWN | UP | DOWN |
| TargetGene | 740 | 713 | 489 | 454 | 454 | 476 | 46 | 84 | 48 | 95 | 48 | 26 |

Table S6. Enrichment analyses of target genes of differentially expressed miRNAs between yaks and cattle.

| Category | P_value | DEG | Gene number | Term | Class |
| --- | --- | --- | --- | --- | --- |
| ko04071 | 0.0000395 | 18 | 54 | Sphingolipid signaling pathway | Environmental Information Processing; Signal transduction |
| ko04932 | 0.0000682 | 13 | 67 | Non-alcoholic fatty liver disease (NAFLD) | Human Diseases; Endocrine and metabolic diseases |
| ko04210 | 0.000641214 | 11 | 32 | Apoptosis | Cellular Processes; Cell growth and death |
| ko04670 | 0.000679095 | 10 | 50 | Leukocyte transendothelial migration | Organismal Systems; Immune system |
| ko05100 | 0.000969873 | 8 | 33 | Bacterial invasion of epithelial cells | Human Diseases; Infectious diseases |
| ko04612 | 0.000978439 | 3 | 13 | Antigen processing and presentation | Organismal Systems; Immune system |
| ko04810 | 0.002900122 | 13 | 89 | Regulation of actin cytoskeleton | Cellular Processes; Cell motility |
| ko00564 | 0.003092563 | 8 | 35 | Glycerophospholipid metabolism | Metabolism; Lipid metabolism |
| ko00190 | 0.004073105 | 8 | 56 | Oxidative phosphorylation | Metabolism; Energy metabolism |
| ko00534 | 0.015962964 | 2 | 9 | Glycosaminoglycan biosynthesis - heparan sulfate / heparin | Metabolism; Glycan biosynthesis and metabolism |

Table S7. Enrichment analyses of target genes of differentially expressed lncRNAs between yaks and cattle.

| Category | P_value | DEG | Gene number | Term | Class |
| --- | --- | --- | --- | --- | --- |
| ko05322 | 0.000000606 | 13 | 92 | Systemic lupus erythematosus | Human Diseases; Immune diseases |
| ko04145 | 0.00000221 | 15 | 82 | Phagosome | Cellular Processes; Transport and catabolism |
| ko00980 | 0.0000263 | 9 | 31 | Metabolism of xenobiotics by cytochrome P450 | Metabolism; Xenobiotics biodegradation and metabolism |
| ko04064 | 0.0000615 | 4 | 44 | NF-kappa B signaling pathway | Environmental Information Processing; Signal transduction |
| ko00983 | 0.0000777 | 7 | 27 | Drug metabolism - other enzymes | Metabolism; Xenobiotics biodegradation and metabolism |
| ko00982 | 0.000121514 | 8 | 28 | Drug metabolism - cytochrome P450 | Metabolism; Xenobiotics biodegradation and metabolism |
| ko00830 | 0.000206776 | 8 | 29 | Retinol metabolism | Metabolism; Metabolism of cofactors and vitamins |
| ko00053 | 0.000476734 | 5 | 11 | Ascorbate and aldarate metabolism | Metabolism; Carbohydrate metabolism |
| ko00140 | 0.000594528 | 6 | 27 | Steroid hormone biosynthesis | Metabolism; Lipid metabolism |
| ko00040 | 0.003093111 | 5 | 17 | Pentose and glucuronate interconversions | Metabolism; Carbohydrate metabolism |

Table S8. Enrichment analyses of target genes of differentially expressed miRNAs among the yaks living at three different altitudes.

| Category | P_value | DEG | Gene number | Term | Class |
| --- | --- | --- | --- | --- | --- |
| ko04071 | 0.001328486 | 13 | 54 | Sphingolipid signaling pathway | Environmental Information Processing; Signal transduction |
| ko04115 | 0.002438243 | 8 | 29 | p53 signaling pathway | Cellular Processes; Cell growth and death |
| ko04931 | 0.002755061 | 10 | 36 | Insulin resistance | Human Diseases; Endocrine and metabolic diseases |
| ko05016 | 0.003142747 | 14 | 87 | Huntington's disease | Human Diseases; Neurodegenerative diseases |
| ko04210 | 0.005362535 | 5 | 32 | Apoptosis | Cellular Processes; Cell growth and death |
| ko04514 | 0.010503853 | 6 | 41 | Cell adhesion molecules (CAMs) | Environmental Information Processing; Signaling molecules and interaction |
| ko04660 | 0.013718435 | 5 | 47 | T cell receptor signaling pathway | Organismal Systems; Immune system |
| ko04670 | 0.017085138 | 6 | 50 | Leukocyte transendothelial migration | Organismal Systems; Immune system |
| ko04068 | 0.022395607 | 5 | 53 | FoxO signaling pathway | Environmental Information Processing; Signal transduction |
| ko04640 | 0.027902204 | 4 | 28 | Hematopoietic cell lineage | Organismal Systems; Immune system |

Table S9. Enrichment analyses of target genes of differentially expressed lncRNAs among the yaks living at three different altitudes.

| Category | P_value | DEG | Gene number | Term | Class |
| --- | --- | --- | --- | --- | --- |
| ko04064 | 0.000514292 | 3 | 44 | NF-kappa B signaling pathway | Environmental Information Processing; Signal transduction |
| ko04520 | 0.000949994 | 5 | 39 | Adherens junction | Cellular Processes; Cellular commiunity |
| ko04530 | 0.006139646 | 3 | 64 | Tight junction | Cellular Processes; Cellular commiunity |
| ko04540 | 0.006528338 | 3 | 50 | Gap junction | Cellular Processes; Cellular commiunity |
| ko04972 | 0.006829934 | 2 | 41 | Pancreatic secretion | Organismal Systems; Digestive system |
| ko04260 | 0.007128511 | 2 | 44 | Cardiac muscle contraction | Organismal Systems; Circulatory system |
| ko04924 | 0.008630812 | 2 | 42 | Renin secretion | Organismal Systems; Endocrine system |
| ko04710 | 0.010056346 | 1 | 12 | Circadian rhythm | Organismal Systems; Environmental adaptation |
| ko04620 | 0.010540171 | 2 | 41 | Toll-like receptor signaling pathway | Organismal Systems; Immune system |
| ko04610 | 0.019916594 | 2 | 43 | Complement and coagulation cascades | Organismal Systems; Immune system |

Table S10. Gene ontology (GO) classifications of differentially expressed genes (DEGs) between yaks and cattle.

| C | Category | Gene Ontology term Top10 | DEGsfrequency | P value |
| --- | --- | --- | --- | --- |
| Biological process | GO:0000038  GO:0006625  GO:0001676  GO:0000038  GO:0000038  GO:0006625  GO:0001676 | very long-chain fatty acid metabolic process  protein targeting to peroxisome  long-chain fatty acid metabolic process  very long-chain fatty acid metabolic process  very long-chain fatty acid metabolic process  protein targeting to peroxisome  long-chain fatty acid metabolic process | 0.60 | 0.000102  0.003059  0.006933  0.005139  0.000101  0.003048  0.007044 |
|  |  |  | 0.50 |  |
|  |  |  | 0.33 |  |
|  |  |  | 0.40 |  |
|  |  |  | 0.60 |  |
|  |  |  | 0.50 |  |
|  |  |  | 0.33 |  |
| Cellular component | GO:0030425  GO:0030173 | dendrite  integral component of Golgi membrane | 0.14 | 0.00319  0.007211 |
|  |  |  | 1.00 |  |
| Molecular function | GO:0004969 | histamine receptor activity | 1.00 | 0.007211 |

Table S11. Gene ontology (GO) classifications of differentially expressed genes (DEGs) among yaks living at three different altitudes.

|  | Category | Gene Ontology term Top10 | DEGsfrequency | P value |
| --- | --- | --- | --- | --- |
| Biological process | GO:2000401  GO:0002377  GO:0006956  GO:0030449  GO:0038096  GO:0050900  GO:0038095  GO:0006898 | regulation of lymphocyte migration  immunoglobulin production  complement activation  regulation of complement activation  Fc-gamma receptor signaling pathway involved in phagocytosis  leukocyte migration  Fc-epsilon receptor signaling pathway  receptor-mediated endocytosis | 1.00  0.50  0.50  0.50  0.50  0.50  0.33  0.33 | 0.001065  0.001004  0.001004  0.001004  0.001004  0.001004  0.001452  0.001744 |
| Cellular component | GO:0072562 | blood microparticle | 0.50 | 0.001004 |
| Molecular function | GO:0004252 | serine-type endopeptidase activity | 0.50 | 0.001004 |

Table S12. Primers used to detect mRNAs from the lungs of yaks by RT-qPCR.

| Genes | GenBank accession No. | Primer sequences (5'-3') | Annealing temperature/℃ | Product size/bp |
| --- | --- | --- | --- | --- |
| CALM1 | XM_005888605.2 | F: GCACCATCACAACCAAGGAAC | 57.7 | 176 |
|  |  | R: CTCTTCACTGTCGGTGTCTTTCA |  |  |
| FNBP1L | XM_005894694.1 | F: AAGAGCCAAGGTTTACTTCGTGT | 57.8 | 86 |
|  |  | R: TGCTACTACTTCTCGCTGTCCTG |  |  |
| MCRS1 | XM_014478414.1 | F: TTCCGACGCAGAGGACTTG | 57.5 | 150 |
|  |  | R: TCTACCAGCACCTGCCACTTAT |  |  |
| FOXP4 | XM_005896443.2 | F: AGGATGTTCGCCTATTTCCG | 57.4 | 140 |
|  |  | R: TTCTGGTACTCCCGCTCGTC |  |  |
| KLHL20 | XM_014476724.1 | F: GGACAAGACCACGGAAACCTA | 56.7 | 127 |
|  |  | R: GCTACCATTCGCCATTCATTAG |  |  |
| RBM15 | XM_005891280.2 | F: AGTGGAGGACGGATTGTTTCA | 56.6 | 109 |
|  |  | R: GTTCACAAAGGCTACTCGCTCA |  |  |
| ANKRD17 | XM_005891242.2 | F: TGTTAGTTGTGCGTTGGATGAA | 54.8 | 109 |
|  |  | R: TGAACAGGCTTCTGCCAAACT |  |  |
| β-actin(yak)  β-actin(cattle) | DQ838049.1  DQ066897.1 | F: ACACGGTGCCCATCTACGA  R: CCTTGATGTCACGGACGATTT  F: TCCGTGACATCAAGGAGAAGC  R: CAGGAAGGAAGGCTGGAAGA | 56.4  57.5 | 159  176 |

Table S13. Primers used to detect lncRNAs from the lungs of yaks by RT-qPCR.

| lncRNA | GenBank accession No. | Primer sequences (5'-3') | Annealing temperature/℃ | Product size/bp |
| --- | --- | --- | --- | --- |
| LOC106701264 | XR_001351744.1 | F: CAGTCCCTTCTTTCCCCTCC | 59.5 | 182 |
|  |  | R: CCAGAGCCATCAAGGTGAAGA |  |  |
| LOC106700864 | XR_001351478.1 | F: TTTCGCTTGTGACGGTGGT | 55.2 | 181 |
|  |  | R: TGCCGCATCACATTCTTCTC |  |  |
| LOC106700715 | XR_001351364.1 | F: AAATCCCAGAAACCAGGTAAGG | 55.8 | 163 |
|  |  | R: AGTTGAAAGAATGGCTGTTGTAGTG |  |  |
| LOC106701327 | XR_001351797.1 | F: GGTCCAGTCTGTCCACCTTTG | 59.5 | 120 |
|  |  | R: CAAGCCTCCTCAGCCTCATC |  |  |
| LOC106701047 | XR_001351599.1 | F: ACAACTAAGCCAACAACAGCAAC | 56 | 89 |
|  |  | R: TCTTCCTGTCTCCTACATCTCCC |  |  |
